# Time-resolved universal temperature measurements using NaYF_4_:Er^3+^,Yb^3+^ upconverting nanoparticles in an electrospray jet

**DOI:** 10.3762/bjnano.9.270

**Published:** 2018-11-21

**Authors:** Kristina Shrestha, Arwa A Alaulamie, Ali Rafiei Miandashti, Hugh H Richardson

**Affiliations:** 1 Department of Chemistry and Biochemistry, Ohio University, Athens, Ohio 45701, USA; 2 Department of Chemistry, College of Science, King Faisal University, Hofuf 31982, Saudi Arabia

**Keywords:** electrospray, microjet, nanothermometry, temperature measurement, time-resolved measurement, upconverting nanoparticles

## Abstract

Hexagonal upconverting nanoparticles (UCNPs) of NaYF_4_:Er^3+^,Yb^3+^ (ca. 300 nm) have been widely used to measure the temperature at the nanoscale using luminescence ratio thermometry. However, several factors limit their applications. For example, changes in the peak shape, mainly is the S-band emission, hinders their ability to be used as a universal temperature sensor. Herein, we introduce a universal calibration protocol for NaYF_4_:Er^3+^,Yb^3+^ upconverting nanoparticles that is robust to environmental changes and gives a precise temperature measurement. We used this new procedure to calculate the temperature profile inside a Taylor cone generated with an electrospray jet. Inside the Taylor cone the fluid velocity increases toward the tip of the cone. A constant acquisition length leads to a decrease in excitation and acquisition time. This decrease in excitation time causes a peak shape change that corrupts the temperature measurement if the entire peak shape is integrated in the calibration. Our universal calibration circumvents this problem and can be used for time-resolved applications. The temperature at the end of the Taylor cone increases due to the creation of a whispering gallery mode cavity with 980 nm excitation. We use time-resolved energy balance equations to support our optical temperature measurements inside the Taylor cone. We believe that the findings of this paper provide a foundation for time-resolved temperature measurements using NaYF_4_:Er^3+^,Yb^3+^ upconverting nanoparticles and can be used to understand temperature-dependent reactions such as protein unfolding inside microjet/microdroplets and microfluidic systems.

## Introduction

There is need and interest to non-invasively image and measure temperatures in complex systems such as in vivo imaging, cellular biological systems and matrices, whole-blood samples, and electrospray jets used in mass spectrometry [1]. Optical thermometers using UCNPs are well suited for these applications because near-IR excitation of the UCNPs minimizes tissue damage [2], is relatively free of background fluorescence [3], and has a high penetration depth [3–4]. Time-resolved temperature measurements using the luminescence intensity ratio (LIR) of UCNPs are rare [5–6]. Here we show that NaYF_4_:Er^3+^,Yb^3+^ UCNPs can provide time-resolved temperature measurements inside an electrospray Taylor cone.

Measuring temperature with nanoscale spatial resolution is not easy. Nanoscale temperature measurements and properties have been modeled [7–9] and measured using lanthanide emission [5,10–15], photothermal properties [16–27], phase transitions [28–29], quantum dot luminescence thermometry [30], and ultrafast pump–probe measurements [31–34]. An optical temperature measurement has the advantage of remote sensing but is diffraction-limited with the spatial uncertainty depending upon the wavelength of the interrogating light. We have shown that the photoluminescence of erbium ions embedded in a wide-band-gap matrix is temperature-dependent [12] and have used the emission to determine the local temperature of optically excited gold nanostructures at an interface. The temperature is determined by measuring the ratio of two green photoluminescence bands where the relative intensities are temperature-dependent and related by a Boltzmann factor. We used this thermal sensor to probe the thermal properties at a solid–water interface and found that a nanoscale object optically heated can superheat water much beyond the boiling point [13].

In this paper, we synthesized and characterized hexagonal UCNPs of NaYF_4_:Er^3+^,Yb^3+^ for temperature measurements. We find that the S-band peak shape changes with 980 nm laser intensity and excitation time. This change of peak shape causes problems when using the luminescence intensity ratio (LIR) to measure temperature unless a calibration is done under identical conditions. We find that this problem can be circumvented by integrating the S-band just over the wavelength range from 536 to 548 nm (the first two peaks in the S-band). The S-band peak shape in this range is more robust to environmental changes, especially changes in the excitation laser light intensity and laser excitation time. The LIR using the reduced S-band integration give temperature measurements with a universal calibration. We applied this new procedure to determine the temperature profile inside a Taylor cone generated with an electrospray jet. The water velocity inside the Taylor cone increases toward the tip of the Taylor cone resulting in a decrease in the illumination time. Unexpectedly, the temperature at the end of the Taylor cone increases due to the formation of a whispering gallery mode (WGM) cavity under 980 nm excitation. We present time-resolved energy balance equations that agree with and support our temperature measurements inside the Taylor cone.

## Results and Discussion

### Characterization of NaYF_4_:Er^3+^,Yb^3+^ upconverting nanoparticles (UCNPs)

**Figure 1:**
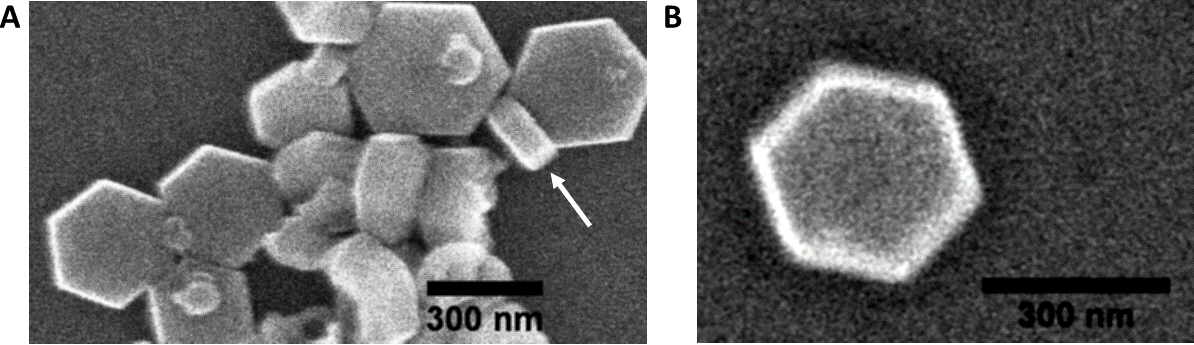
(A) SEM image of synthesized NaYF_4_:Er^3+^,Yb^3+^ UCNPs using thermal decomposition method. (B) An image of a single nanoparticle. The nanoparticle has a hexagonal shape with a diameter of ca. 300 nm and a thickness of ca. 80 nm.

A scanning electron microscopy (SEM) image of a thin film of NaYF_4_:Er^3+^,Yb^3+^ UCNPs drop-cast on a glass coverslip is shown in Figure 1. The UCNPs are relatively uniform in size and shape with an average diameter around 300 nm and a height of around 80 nm. The height (thickness) of the UCNPs is determined by observing particles standing on end in the SEM image (see Figure 1A arrow).

**Figure 2:**
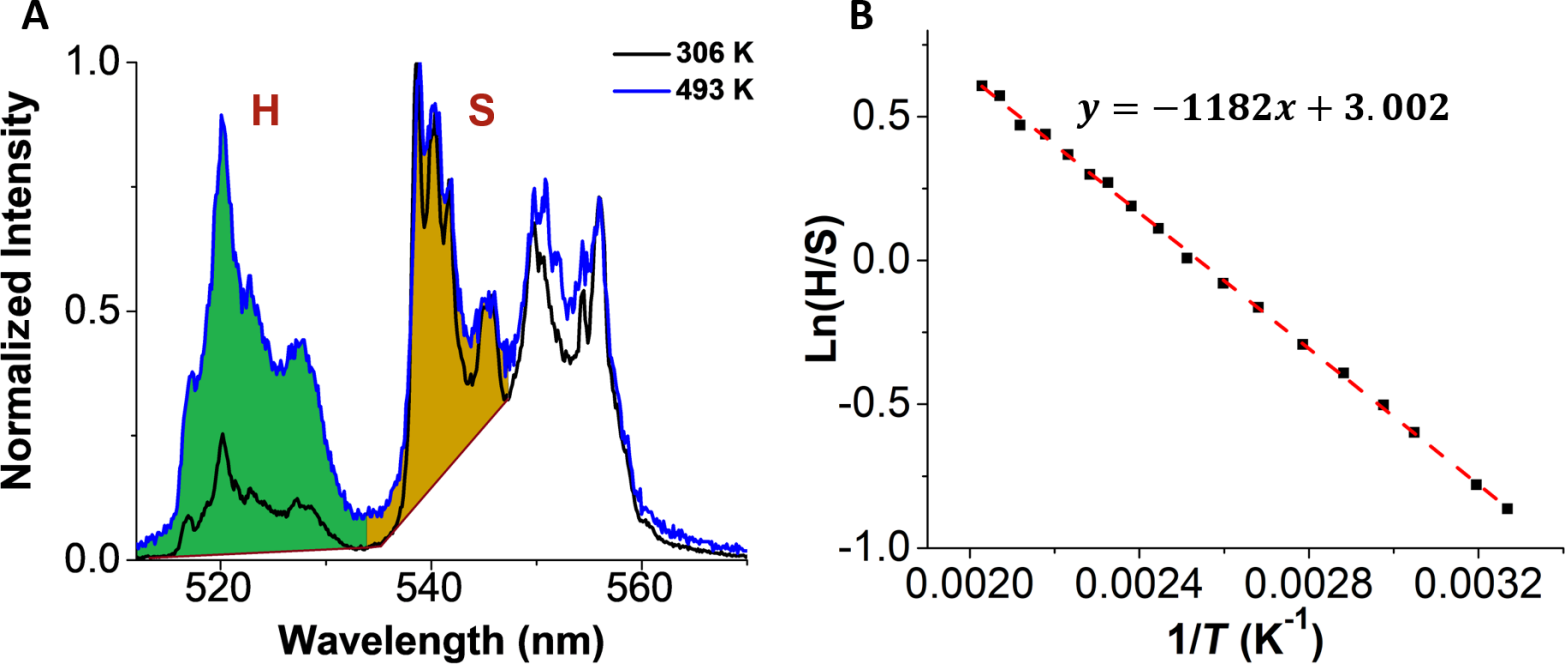
(A) Upconversion emission spectra of UCNPs at different temperatures. A temperature-dependent green emission is measured for a temperature range of 306–493 K. (B) A plot of ln(H/S) vs 1/*T* for calibration of UCNPs. The H- (514–534 nm) and S- (536–548 nm) band area selected for the calibration are shaded in green and brown, respectively, in (A). The slope of the line calculated is −1182 ± 8 K and the intercept is 3.002 ± 0.021.

The optical response from the UCNPs with temperature is shown in Figure 2. The UCNPs are excited with 980 nm laser light and emit in the visible. It has been previously shown that the luminescence intensity ratio (LIR) between the H- and S-band changes with temperature [35]. The LIR is used to calculate the absolute temperature using the Boltzmann relationship. Here we use H-band (514–534 nm) and S-band (536–548 nm) peak areas for LIR. Figure 2A represents the area of H- and S-band of UCNPs shaded in green and brown, respectively. We changed the temperature of a coverslip with UCNPs on it, from 306 to 493K and collected the spectra using an α-300 SNOM Witec microscope with optical-fiber-coupled monochromator. Figure 2B represents the plot of natural log of LIR vs 1/*T* where *T* is the recorded temperature. A linear fit gives a slope value of −1182 ± 8.0 K and the intercept is 3.002 ± 0.021.

**Figure 3:**
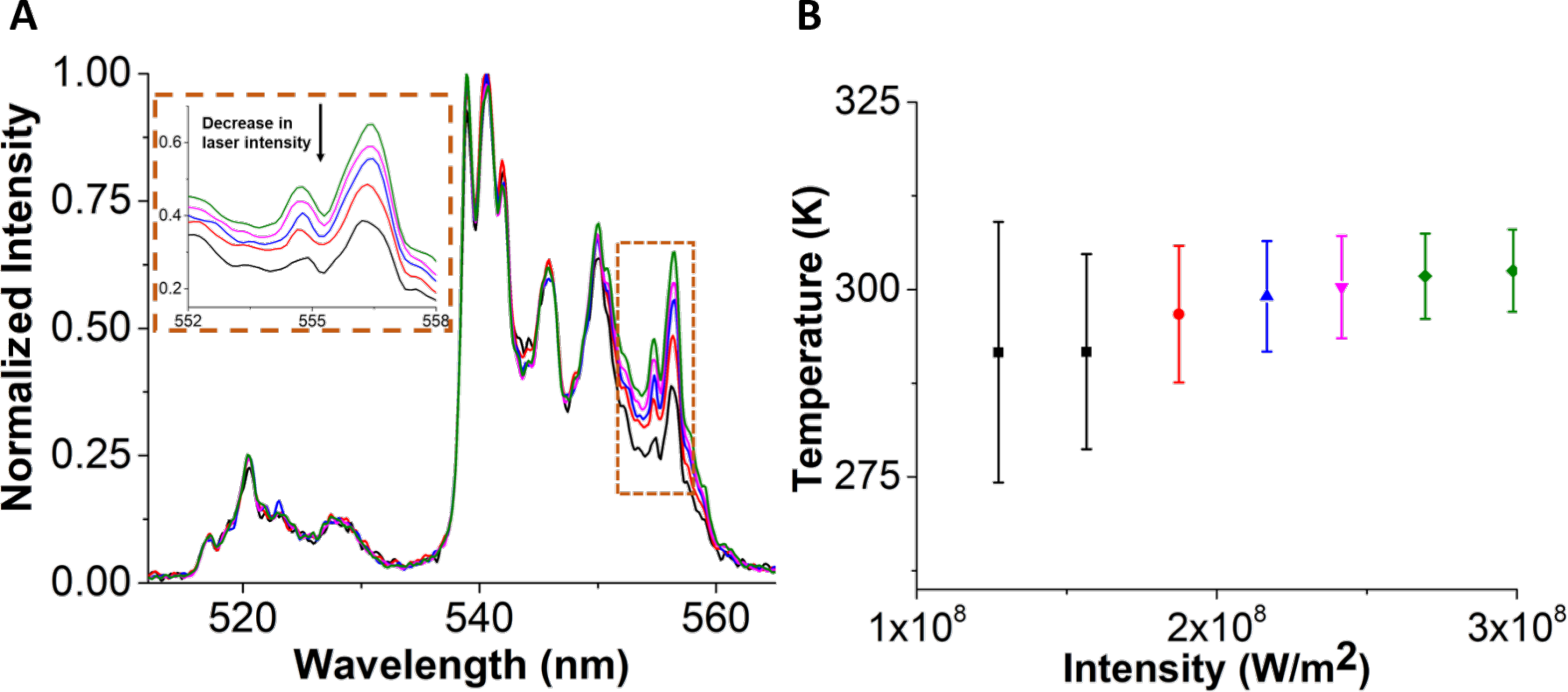
Measurement of temperature of UCNPs at different laser intensities of 980 nm. (A) Photoluminescence spectra of a single cluster of UCNPs of ca. 1 μm size at different laser intensities. With the decrease in intensity, there is also decrease in an integrated area of S-band after 548 nm. The inset shows an enlarged view of a change in peak intensity with respect to laser intensity. (B) A plot of an average temperature plotted as a function of 980 nm laser intensity. The temperature calculated remains constant with a decrease in standard deviation upon an increase in laser intensity. An emission spectrum of a given color in (A) represents a temperature in the plot in (B).

The selection of the peak range is important for an accurate calculation of temperature. Changes in peak shape with external factors such as intensity, excitation time, and surrounding dielectric conditions [36] will corrupt the temperature measurement unless a calibration is made under identical conditions. We observed that the peak shape of the S-band changes with 980 nm laser intensity (Figure 3) and with the 980 nm laser excitation time (see below in Figure 5). Figure 3A shows H- and S-band for a single cluster of ca. 1 μm UCNPs at different 980 nm laser intensities. We noticed that the peak shape changes with a change in laser intensity as shown in Figure 3A (inset). The H-band and partial S-band (536–548 nm) remained invariant with laser intensity change but the lower energy portion of the S-band after 548 nm shows a decrease in peak area with a decrease in laser intensity. This leads us to calibrate our particles using the H-band in the range of 514–534 nm and the S-band in the reduced range of 536–548 nm. This is the method of calibration that was used in Figure 2. The temperature calculated using this new calibration is shown in Figure 3B at different laser intensities. The average temperature remains fairly constant with a decrease in standard deviation upon an increase in laser intensity. The average temperature and standard deviation are calculated from histograms of a temperature–time spectrum that is fitted with a Gaussian as shown in Supporting Information File 1, Figure S1. At a laser intensity of 3.0 × 10^8^ W/m^2^, the standard deviation is 6 K. This gives a noise floor of 4 K·Hz^−1/2^ (see Supporting Information File 1, Table S1) with an integration time of 0.5 s. Interestingly, the peak shape does not change with increasing number of UCNPs excited (see Figure S2 in Supporting Information File 1).

**Figure 4:**
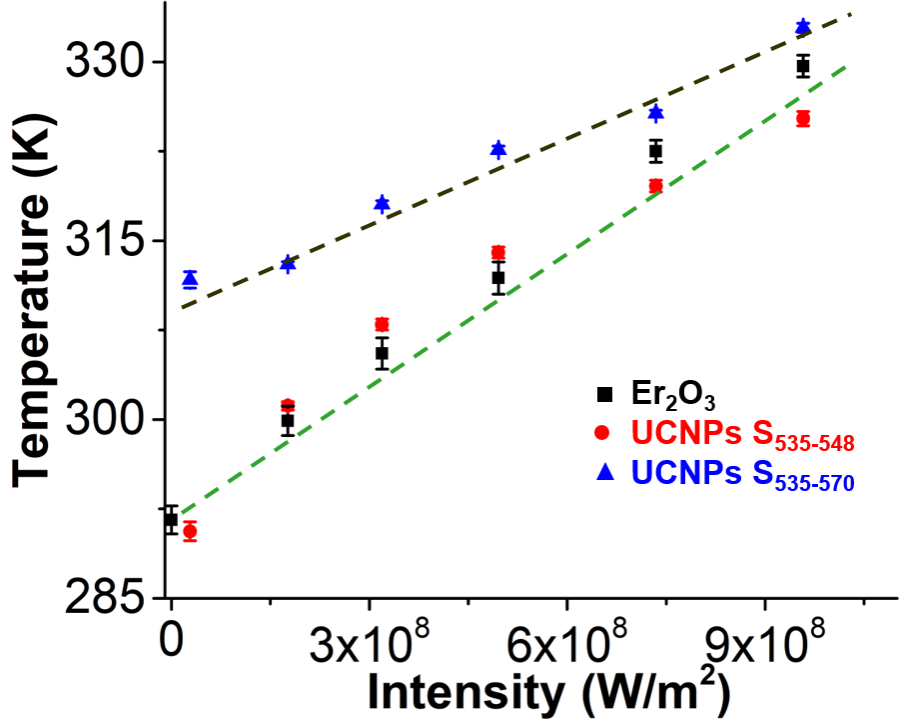
A plot of the temperature of water calculated as a function of the 980 nm laser intensity using two different nanothermometers, namely erbium oxide (Er_2_O_3_) and UCNPs. The temperature of UCNPs is calculated using both the full S-band from 535 to 570 nm and the partial S-band from 535 to 548 nm. The temperature calculated using S_535–548_ is in good agreement with our reference thermal sensor using Er_2_O_3_ nanoparticles [37].

We checked this new calibration using only the first two peaks of the S-band to determine LIR against another reference optical thermometer developed in our lab (Erbium Oxide nanoparticles) [37]. We chose to measure the temperature of water as the 980 nm laser intensity is changed. An additional laser at 532 nm is used to excite the Er_2_O_3_ nanoparticles. The water absorption at 532 nm is significantly lower than at 980 nm and water heating occurs predominately from the 980 nm laser [38]. Figure 4 shows the temperature of water calculated by both UCNPs and Er_2_O_3_ at different 980 nm laser intensities. For UCNPs, we used both full S-band from 535–570 nm and the reduced S-band from 535–548 nm. The plot shows that our reduced S-band calibration method is in good agreement with our reference optical thermometer, while the traditional method is not. Because the 532 nm laser remains constant throughout the experiment, the temperature uncertainty using Er_2_O_3_ nanoparticles remains almost constant even when the 980 nm laser is changed. However, we do observe a decrease in the temperature uncertainty calculated from the emission of the UCNPs because the emission intensity is directly related to the 980 nm laser intensity. The emission spectra for UCNPs and Er_2_O_3_ at different 980 nm laser intensities is shown in Figure S3 of Supporting Information File 1. For Er_2_O_3_ nanoparticles, we observe that the peak shape does not change with increasing 980 nm laser intensity. Figure 4 also shows that if the entire S-band is used to determine the temperature, the temperature is overestimated at low 980 nm laser intensities because of changes in peak shape. This overestimation in temperature converges to the true temperature at high laser intensities. We believe that the new calibration gives accurate temperature measurements even though the excitation laser intensity or excitation time is changed. This is critical for temperature measurements inside a Taylor cone (see below) because the water velocity and excitation time changes at different positions in the Taylor cone.

### Temperature measurements inside the Taylor cone

**Figure 5:**
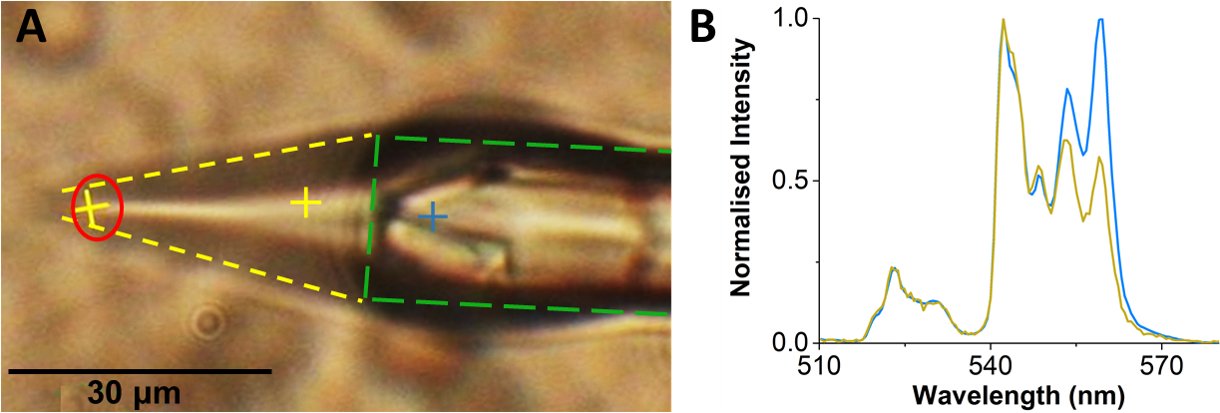
(A) An image showing an electrospray jet of 0.8 pM upconverting solution (yellow lines) from a glass pipette (green lines). The red circle represents the tip of a Taylor cone. (B) Emission spectra of UCNPs at different regions as marked in panel (A) with respective colors. It shows a marked difference in the nature of spectra of UCNPs when they are in a glass pipette or flowing in a Taylor cone.

An image of an electrospray jet of the upconverting solution from a glass pipette is shown in Figure 5A. A yellow dotted line marks a Taylor cone and the green dotted line represents a glass tip with a diameter of ca. 15 μm. Figure 5B shows the comparative emission spectra of UCNPs at different regions of the Taylor cone shown in Figure 5A. The blue spectrum represents the emission for stationary UNCPs adhered to the glass tip and the yellow spectrum for particles moving in the Taylor cone. These normalized spectra clearly show a difference in peak shapes for the S-band that is similar to the peak shape differences observed by varying the 980 nm laser intensity. These peak shape differences appear to be invariant to the first two peaks in the S-band (peaks located at 545 nm and 548 nm). For this reason, all subsequent temperature measurements use only the integrated peak areas for the first two peaks in the S-band.

**Figure 6:**
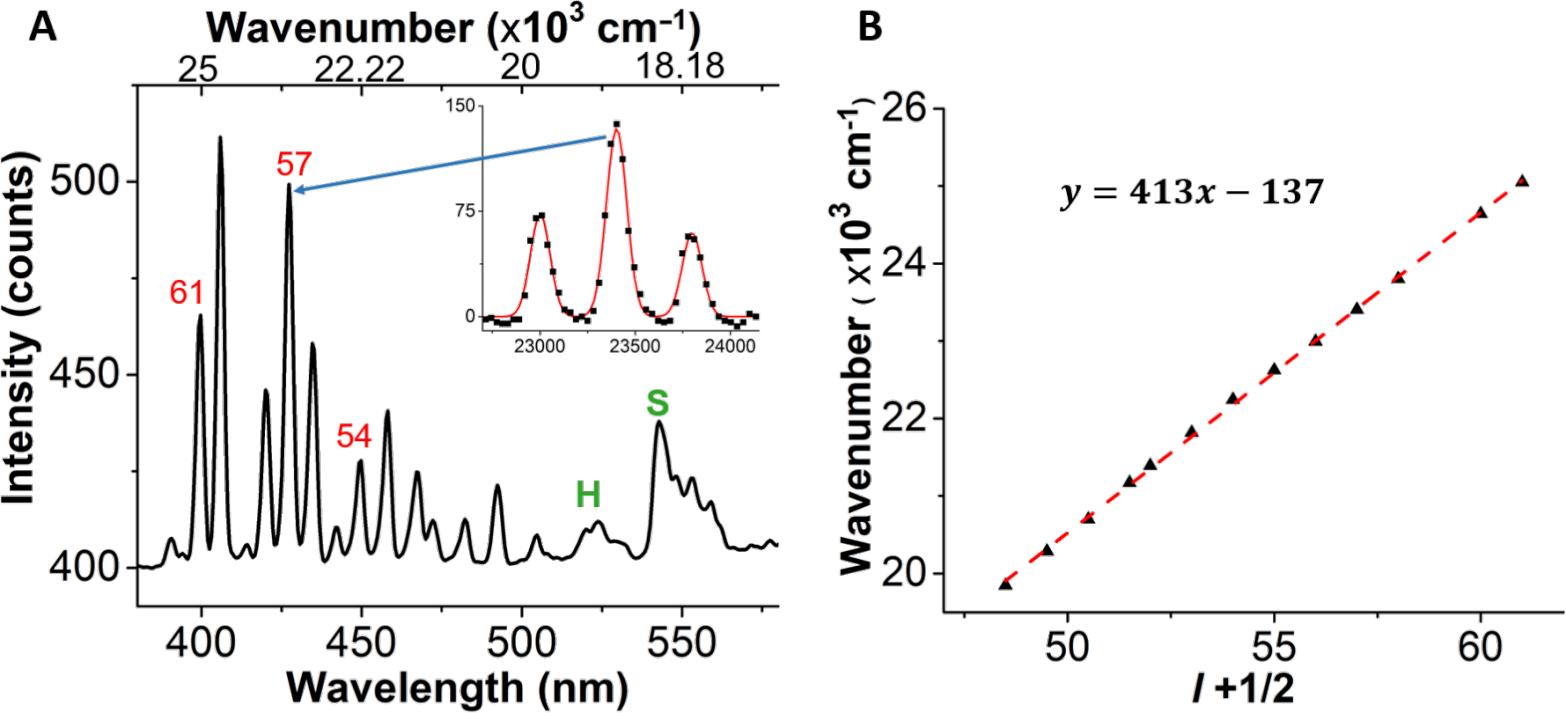
(A) A plot of intensity vs wavelength/wavenumber exhibiting whispering gallery modes at the tip of a Taylor cone indicated by a red circle in Figure 5A. The inset represents a peak profile, fitted with Gaussian to calculate the *Q*-factor. The values in red represent *l* + 1/2 values calculated using the respective peaks. (B) A plot of wavenumber vs *l* + 1/2 with slope 413 ± 3 and intercept of −137 ± 158. The cavity radius is 2.87 ± 0.02 µm.

The radius of the Taylor cone tip is measured spectroscopically by observing the spectral differences in WGMs from a WGM cavity created at the tip. The emission spectrum at the tip compared to the emission spectrum at the beginning of the Taylor cone is presented in Supporting Information File 1, Figure S4. New peaks in the emission spectrum are observed when the 980 nm laser is focused at the tip on the Taylor cone. These new peaks are assigned to WGMs. The emission spectrum at the tip with mode spacing is shown in Figure 6. At the tip of the cone, the jet turns into a spherical droplet that when excited with 980 nm light, creates a WGM cavity. The cavity allows light to circle around the spherical structure and interfere constructively. Figure 6A shows the spectrum at the tip of a cone marked with red circle in Figure 5A. The WGMs appears in a spectral region extending from 400 to 495 nm (20000–25000 cm^−1^) and can be used to calculate the cavity radius using the equation


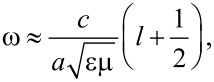


where ω is the angular frequency, *c* is the speed of light, ε and μ are relative permittivity and permeability of water, *l* is an integer, and *a* is the radius of the WGM cavity [39–40]. The plot of wavenumbers of WGM peaks vs as a function of *l* + 1/2 is shown in Figure 6B. The slope from a linear fit is used to calculate the cavity radius of 2.87 ± 0.02 μm. The *Q*-factor of the WGM cavity can be calculated using 
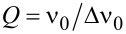
 where ν_0_ is the wavenumber of the peak center and Δν_0_ is the FWHM of the peak [40]. The inset in Figure 6A shows the peak and fit for the three peaks near 425 nm. The *Q*-factor is found to be 165 ± 20.

**Figure 7:**
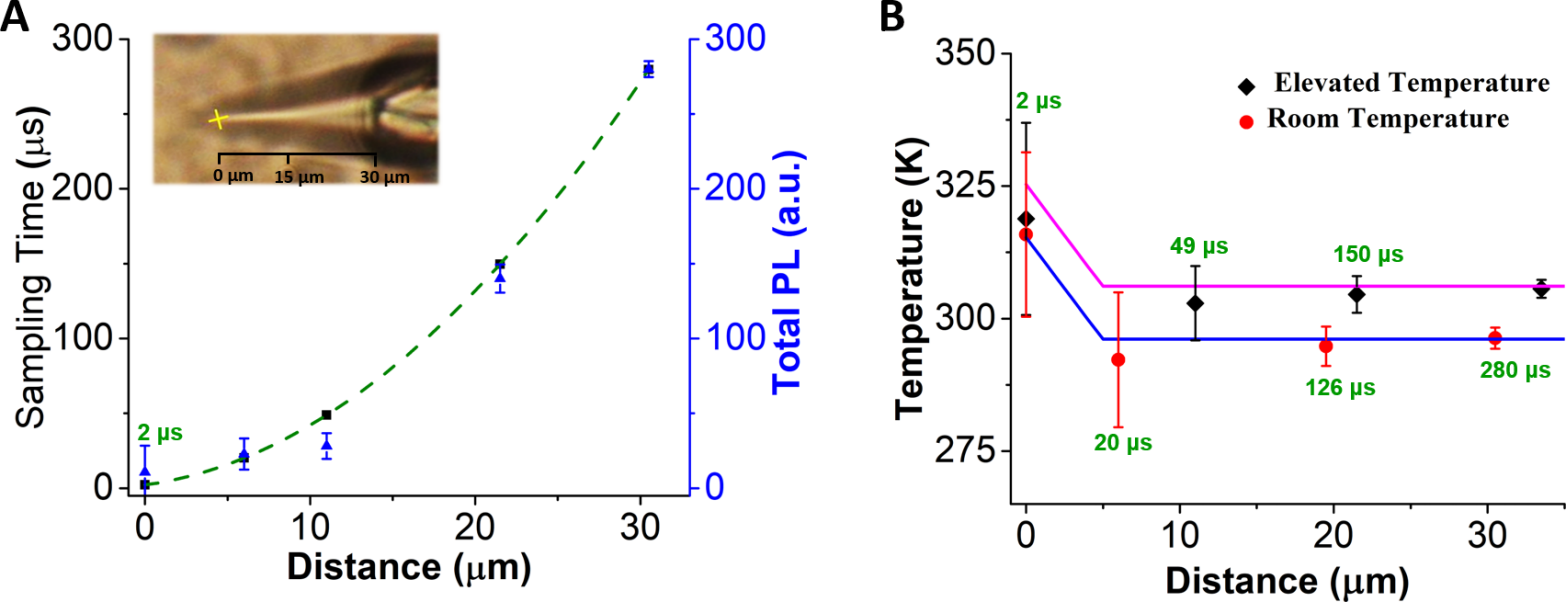
(A) Plot of sampling time and total photoluminescence intensity of UCNPs versus distance from the tip of a Taylor cone (*x*_tip_ = 0). Both sampling time and intensity follow an *r*^2^-dependence along the jet where *r* is the radius of the cross section perpendicular to *x*. Distance *x* and radius *r* are related by *r* = *x* tanθ where θ is the Taylor cone angle. The sampling time reduces to 2 μs at the tip. (B) A plot of temperature calculated along the trajectory of a Taylor cone at room temperature (296 K) and at elevated temperature (306 K). The sampling time at a given distance is indicated in the figure.

The water velocity changes inside the Taylor cone with the highest velocity at the tip of the cone [41]. As the water and particles move toward the tip in an electrospray jet, the velocity increases and the time during which water is optically heated decreases. Consequently, the UCNPs illumination and sampling interval decreases. The water velocity can be calculated using 
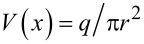
 where *q* is the flow rate of the solution, *r* is the radius of a cylinder with height *h*_w_ (FWHM of the laser spot area) at a given distance *x* from the tip of the Taylor cone. Velocity is related to the sampling time (τ) as


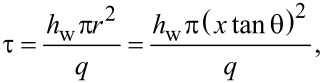


where θ is the Taylor cone angle of 20°. The emission intensity has an *r*^2^-dependence on the distance *x*, similar to the sampling time. Both the emission intensity and the sampling rtime decrease closer to the tip of the Taylor cone. This effect is shown in Figure 7A where the normalized total emission intensity and sampling time are plotted as functions of the distance *x* from the tip of the Taylor cone. The green line in Figure 7A is an *r*^2^-fit to the total emission intensity and sampling time.

Our analysis of the temperature change inside the Taylor cone begins with the energy balance equation shown in Equation 1 [7]. In this equation *m_i_* and *C*_p,_*_i_* are the mass and heat capacity components of the system, *T* is temperature, *t* is time, *Q*_I_ and *Q*_ext_ are the rates of energy supplied and flowing out of the system respectively. The energy supplied to the system is by light absorption of water at 980 nm and is given by Equation 2. *P*_0_ is the laser power at 980 nm (0.27 W), *σ*_abs_ is the absorption cross section of water at 980 nm (3 × 10^−28^ m^2^/molecule) [38], *N*_w_ is the number density of water (3.33 × 10^28^ molecules/m^3^).

[1]
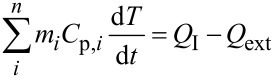


[2]
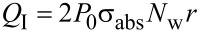


The rate of heat loss is given by Equation 3 where *h* and *S* are the heat-transfer coefficient and surface concentration of water perpendicular to the direction of heat conduction.

[3]
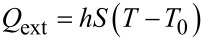


Equation 1 can be simplified to 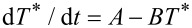 where the rate of energy absorption, *A*, is


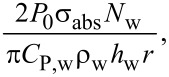


and the rate of heat release, *B*, depends upon the size of the heated object [42–43]:


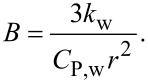


Here, T^*^ is equal to the temperature change (*T* − *T*_0_), *k*_w_ is the thermal conductivity of water (0.6 W·m^−1^·K^−1^), *C*_P,w_ is the heat capacity of water (4.18 J·g^−1^·K^−1^) and ρ_w_ is the density of water. This simplified equation, when solved, gives the solutions presented in Equation 4 and Equation 5. Equation 4 gives the solution when there is no energy input into the system (*A* = 0), and Equation 5 is the solution when energy is supplied to the system (A ≠ 0). When the system reaches a steady state, the temperature change is equal to *A*/*B*. The steady-state temperature is given by Equation 6.

[4]
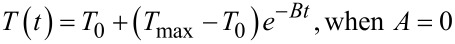


[5]
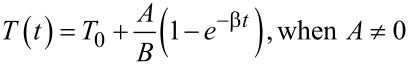


[6]
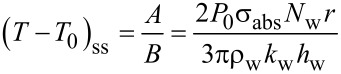


The temperature when the system is not in a steady state (short pulse light excitation) is given by Equation 7. Here, the approximation *e*^−^*^Bt^* ≈ 1 is used with substitution of the sampling interval for time:

[7]
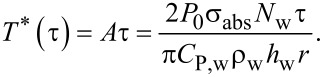


Combining the sampling time of


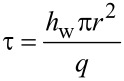


with Equation 7 gives Equation 8:

[8]
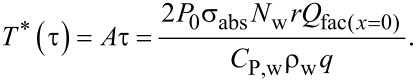


In Equation 8 the temperature directly depends on *r*, and because *r* decreases towards the tip of the Taylor cone, the temperature is expected to decrease. Unexpectedly, light excitation at the tip of the Taylor cone sets up a WGM cavity increasing the optical path length by the *Q*-factor of the cavity [39]. The increased path length results in more light absorption at the tip of the Taylor cone and an increased instead of a decreased temperature as expected. This change of path length can be accounted for by multiplying the expected temperature change by the *Q*-factor of the WGM cavity over the distance range in which the WGM is observed (*x* < 5 μm). The integrated areas for the WGMs peaks plotted as functions of the distance are shown in Supporting Information File 1 (Figure S4). Our model fit is shown in Figure 7B as the solid blue (room temperature) and purple line (temperature raised 10 K with external heater). The model fit uses a *Q*-factor of 165. This factor is in agreement with the *Q*-factor estimated from the peak position and width (see Figure 6A).

## Conclusion

In this paper we introduce a universal calibration protocol for NaYF_4_:Er^3+^,Yb^3+^ UCNPs that is robust to environmental changes and gives a more precise temperature measurement. We applied this new procedure to determine the temperature profile inside a Taylor cone generated with an electrospray jet. Inside the Taylor cone the fluid velocity increases toward the tip of the cone. A constant acquisition length results in a decrease in excitation and acquisition time. This decrease in excitation time causes a peak shape change that corrupts the temperature measurement if the entire peak shape is used in the calibration. Our universal calibration circumvents this problem and can be used for time-resolved applications. The temperature at the end of the Taylor cone increases due to the creation of a WGM cavity with 980 nm excitation. We use time-resolved energy balance equations to support our optical temperature measurements inside the Taylor cone. We believe that the findings of this paper provide a foundation for time-resolved temperature measurements using NaYF_4_:Er^3+^,Yb^3+^ UCNPs and can be used to understand temperature dependent reactions like protein unfolding phenomenon inside the microjet/microdroplets.

## Experimental

### Synthesis of NaYF_4_:Er^3+^,Yb^3+^ upconverting nanoparticles

The UNCPs were synthesized using the thermal decomposition method. Briefly, sodium trifluoroacetate was added to a mixture of oxides of ytterbium, yttrium, and erbium and decomposed in oleic acid and octadecane solvent. UNCPs synthesized were hydrophobic in nature, due to a presence of oleic acid on its surface, which was removed by adding EDTA. The synthesized particles are of an average size of 300 nm.

### Experimental setup for temperature measurements in an electrospray jet

**Figure 8:**
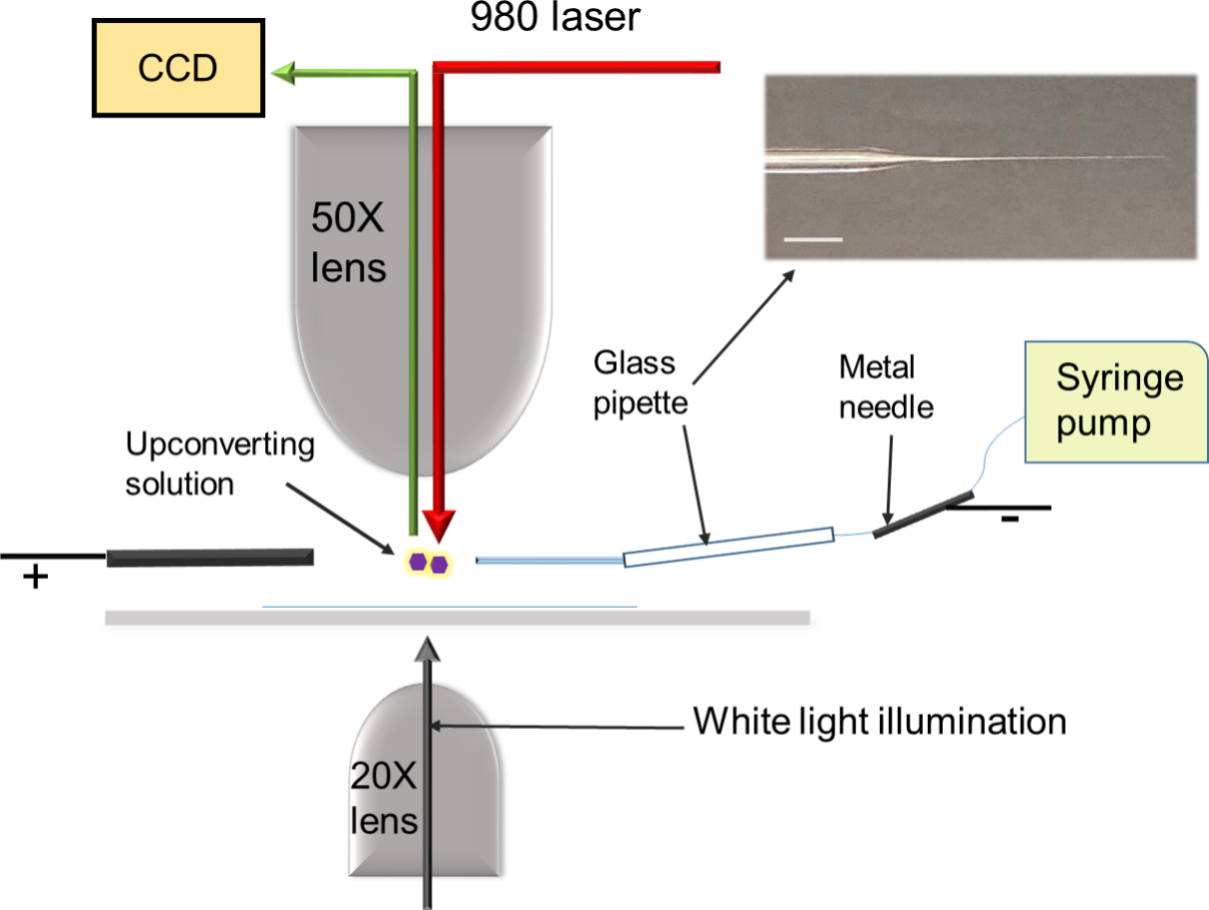
A schematic illustration for a setup of temperature measurement in a Taylor cone, formed by electrospray of an upconverting solution. A 980 nm laser illuminates from the top with a 50× lens. A white light illuminates from the bottom using a 20× lens for imaging. The image on the top right shows a glass capillary pulled to form a ca. 15 μm pipette. The scale bar is 2 mm.

An experimental setup for temperature measurement in an electrospray, using UCNPs as thermal sensors, is shown in Figure 8. A 980 nm laser is reflected off a dichroic mirror onto the electrospray region (using 50× (NA 0.55) objective) and the sample is illuminated from underneath by white light. The intensity of 980 nm laser is held constant throughout the experiment. A voltage of approximately 1.8 kV is applied via a metal needle connected to a glass pipette (approximately 15 μm diameter at the tip) containing a solution of 0.8 pM UCNPs in water. The solution is made ionic by adding a small amount of formic acid. The emission from UCNPs is collected back from same 50× lens via an optical fiber to CCD. All measurements were conducted using a WITec α-SNOM300s microscope.

## Supporting Information

File 1 Additional material.
