# Supplementary material for: Time-resolved universal temperature measurements using NaYF4:Er3+,Yb3+ upconverting nanoparticles in an electrospray jet
Source: Beilstein J Nanotechnol. 2018 Nov 21;9:2916–24. doi: 10.3762/bjnano.9.270 (PMC6278772; doi:10.3762/bjnano.9.270)
Supplement: File 1 — Additional material. [file Beilstein_J_Nanotechnol-09-2916-s001.pdf]

BEILSTEIN JOURNAL OF NANOTECHNOLOGY

# Supporting Information

for

# Time-resolved universal temperature measurements using $NaYF_4$:$Er^{3+}$,$Yb^{3+}$ upconverting nanoparticles in an electrospray jet

Kristina Shrestha, Arwa A. Alaulamie, Ali Rafiei Miandashti and Hugh H. Richardson

*Beilstein J. Nanotechnol.* **2018,** *9,* 2916–2924. doi:10.3762/bjnano.9.270

# Additional material

License and Terms: This is a supporting information file under the terms of the Creative Commons Attribution License (http://creativecommons.org/licenses/by/4.0). Please note that the reuse, redistribution and reproduction in particular requires that the authors and source are credited.
The license is subject to the *Beilstein Journal of Nanotechnology* terms and conditions: (https://www.beilstein-journals.org/bjnano)

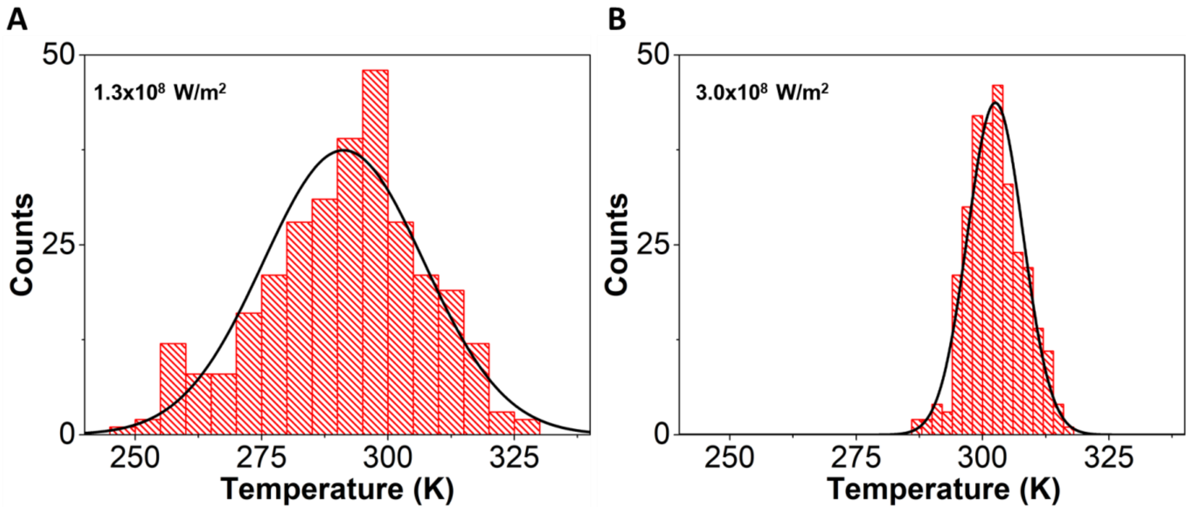


**Figure S1:** (A,B) Histograms of temperatures generated from temperature – time spectrum of upconverting particles cluster of ≈1 μm in size at $1.3 \times 10^8$ and $3.0 \times 10^8$ W/m$^2$ 980 nm laser intensities respectively. They show a normal distribution and are fitted with a Gaussian to calculate an average and standard error of the measurements.

The effect of intensities on the noise floor is demonstrated in Table S1. At a certain intensity, the noise floor can be calculated as the product of standard deviation of temperature and the square root of integration time. The integration time is kept constant at 0.5 s. As the intensity goes higher, the standard deviation goes lower and thus the noise floor.

**Table S1:** Calculation of noise floor at different intensities of laser power with an integration time of 0.5 s.

| Intensity (W/m$^2$) | Temperature (K) | St Dev (K) | Noise floor (K·Hz$^{-1/2}$) |
|---|---|---|---|
| $1.3 \times 10^8$ | 292 | 17 | 12 |
| $1.6 \times 10^8$ | 292 | 13 | 9 |
| $1.9 \times 10^8$ | 297 | 9 | 6 |
| $2.2 \times 10^8$ | 299 | 7 | 5 |
| $2.4 \times 10^8$ | 300 | 7 | 5 |
| $2.7 \times 10^8$ | 302 | 6 | 4 |
| $3.0 \times 10^8$ | 302 | 6 | 4 |

Because there is a change in peak shape with laser intensity, it is necessary to see if there is a change in peak shape with the number of UCNPs excited. Figure S2 shows the effect of the number of upconverting particles (UCNPs) excited on our spectral shape and temperature measurement. Figure S2 (B) shows the emission spectra of different clusters of UCNPs as labelled in Figure S2 (A). The emission signal from the UCNPs, when illuminated with a constant intensity of 980 nm light, is expected to increase with the number of nanoparticles excited. There is a 50-fold increase in emission signal from cluster 7 to cluster 1, suggesting that there is a corresponding increase in the number of UCNPs excited between clusters 7 and cluster 1. But the spectral change is negligible with change in the number of UCNPs. Figure S2 (C) reveals that the average temperature change remains constant within error. A 50-fold increase in signal should reduce the standard deviation by $\sqrt{50} \approx 7$. This reduction is

observed when comparing the standard deviation between cluster 7 and cluster 1. It shows that the number of particles doesn't have an effect on spectral shape and thus an average temperature but it effects the standard deviation of a temperature measurement.

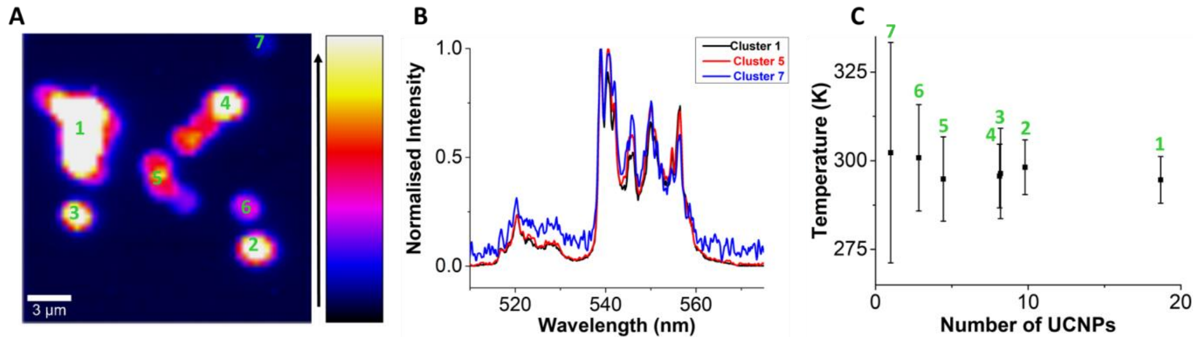


**Figure S2:** The temperature calculated using different clusters of upconverting nanoparticles (UCNPs) at a constant laser intensity. (A) An image scan of different sizes UCNPs clusters. The color scale maps an increase in intensity of the clusters which are directly related with a number of UCNPs in a cluster. (B) Emission Spectra of different clusters labelled as in figure (A) collected at 0.15 integration time. The spectra are similar in shape but signal to noise ratio decreases with the decrease in number of UCNPs. (C) A plot of temperature for of different clusters of UCNPs labelled as in figure (A). The number of UCNPs are calculated relative to cluster 7. They show relatively same temperature but the standard deviation is larger for clusters with lower number of UCNPs.

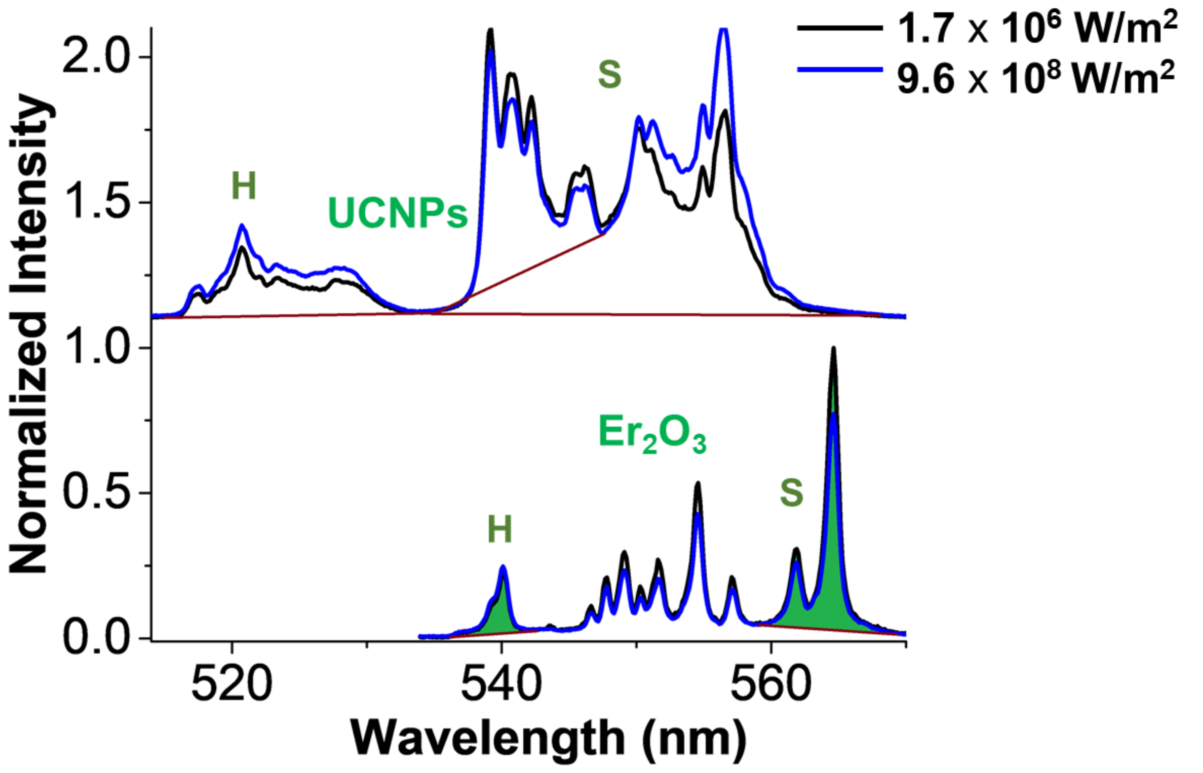


**Figure S3:** Emission spectra of upconverting particles (UCNPs) and Erbium oxide ($Er_2O_3$) at different laser intensities of 980 nm. The emission intensities are normalized (0-1) for comparison at different laser intensities. Red lines are the baselines used for H and S band selection. For $Er_2O_3$, an additional 532 nm laser is illuminated at a constant intensity of $9 \times 10^8$ W/m$^2$. The spectra are displaced for clarity.

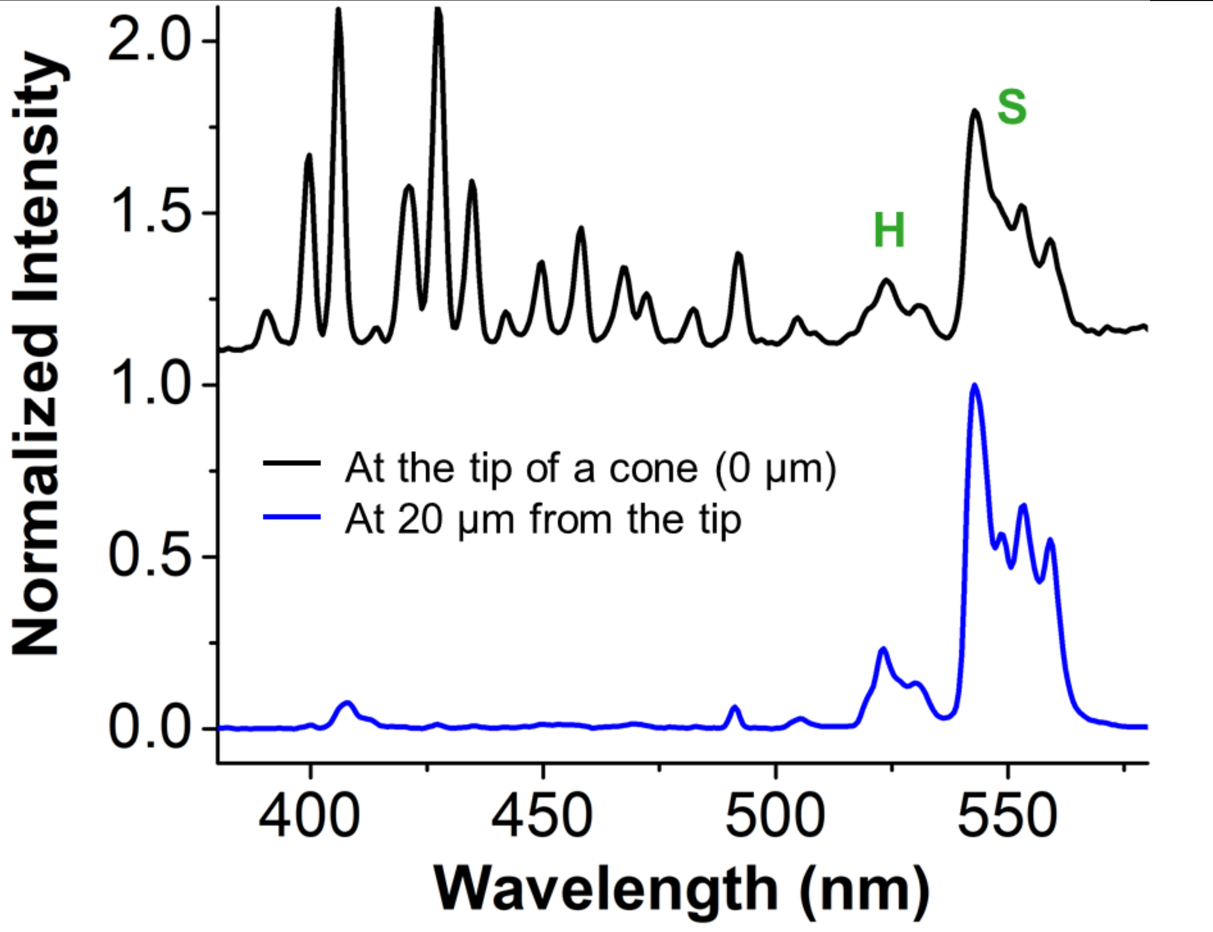


**Figure S4:** Emission spectra at different region of an electrospray jet. The black spectrum shows a spectrum at the tip where a droplet is formed. It is marked by the appearance of whispering gallery modes (WGMs). The blue spectrum is 20 μm from the tip. The spectra are displaced for clarity.
